# Electrochemical data of single and hybrid sol–gel coating precursors for aluminum alloy corrosion protection in 3.5% NaCl

**DOI:** 10.1016/j.dib.2019.01.029

**Published:** 2019-01-18

**Authors:** M. Hazwan Hussin

**Affiliations:** Materials Technology Research Group (MaTReC), School of Chemical Sciences, Universiti Sains Malaysia, 11800 Minden, Penang, Malaysia

## Abstract

The anti-corrosion performances of single(TEOS) and hybrid (APTES-TEOS) sol–gel coatings on Al alloy samples exposed to 3.5 wt% NaCl were evaluated employing electrochemical techniques such as electrochemical impedance spectroscopy (EIS) and potentiodynamic polarization. The data acquired using the three corrosion analysis techniques were in accordance with each other where hybrid sol–gel coating offered the lowest corrosion rate and current density in comparison to the single precursor silanol coating. Tafel curves suggested that the hybrid silane coatings mitigate both the anodic and cathodic reactions simultaneously (mixed type inhibitor). These techniques justified that incorporation of hybrid sol–gel improved the Al corrosion protection performance considerably.

## Specifications table

**Table:** 

Subject area	*Chemistry*
More specific subject area	*Electrochemistry, Corrosion*
Type of data	*Figure, table*
How data was acquired	*GAMRY Reference600 Potentiostats. All data were analyzed using EChem Analyst software.*
Data format	*Analyzed*
Experimental factors	*The data was obtained at a fixed environment (in 3.5* *wt% NaCl solution), fixed polarization potentials and fixed frequency range for EIS analysis.*
Experimental features	*The data was obtained from the corrosion of aluminium alloy coated with single and/or hybrid sol-gel precursors.*
Data source location	*School of Chemical Sciences, Universiti Sains Malaysia, Penang, Malaysia*
Data accessibility	*Data are available here in this article*
Related research article	*E. Alibakhshi, M. Akbarian, M. Ramezanzadeh, B. Ramezanzadeh, M. Mahdavian. Evaluation of the corrosion protection performance of mild steel coated with hybrid sol–gel silane coating in 3.5* *wt% NaCl solution. Progress in Organic Coatings (2018) 123, 190–200.*

## Value of the data

• The given data describe the inhibitive performance of single and hybrid sol–gel precursors on aluminium alloy corrosion in 3.5 wt% NaCl environment.
• The given data will show author in the field of corrosion science the effect of single and hybrid precursors on providing corrosion protection for aluminium corrosion.
• The data obtained could be used to check the correlation of precursors molar ratio on the corrosion of aluminum alloy in 3.5 wt% NaCl environment.
• The data can be used to examine the relationship between the process variable as it affect the nature of inhibition of metals.

## 1 Data

### 1.1 Electrochemical impedance spectroscopy (EIS)

**Fig. 1:**
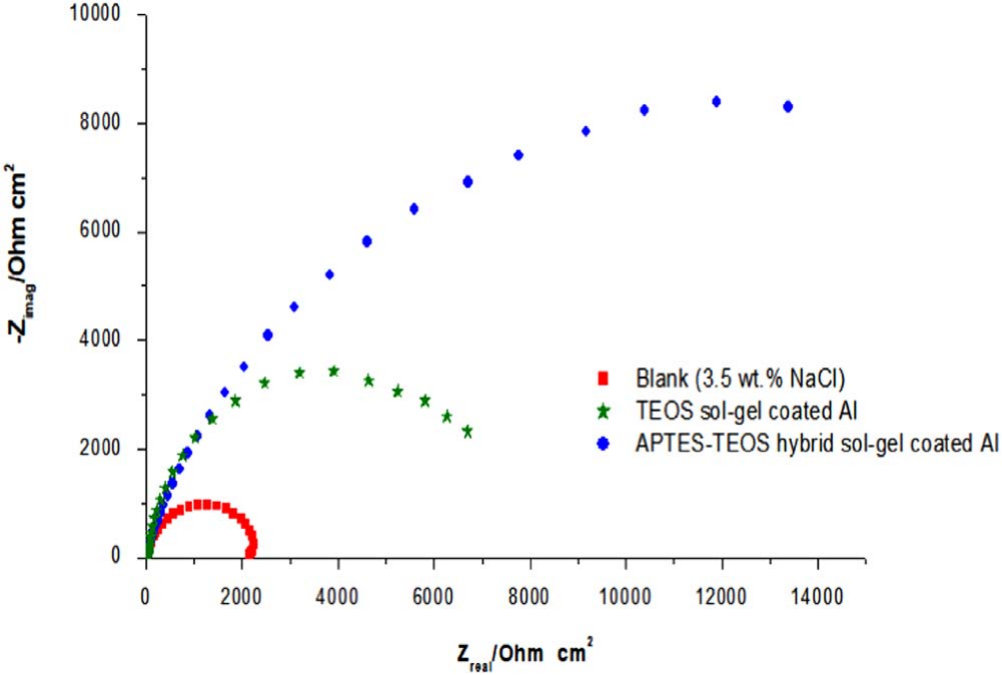
Nyquist plots for bare Al, single (TEOS) and hybrid (APTES-TEOS) sol–gel coatings at 303 K.

**Fig. 2:**
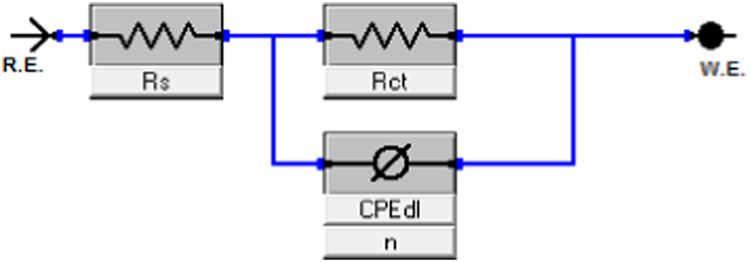
Equivalent electrical circuit to model EIS data of bare Al.

**Fig. 3:**
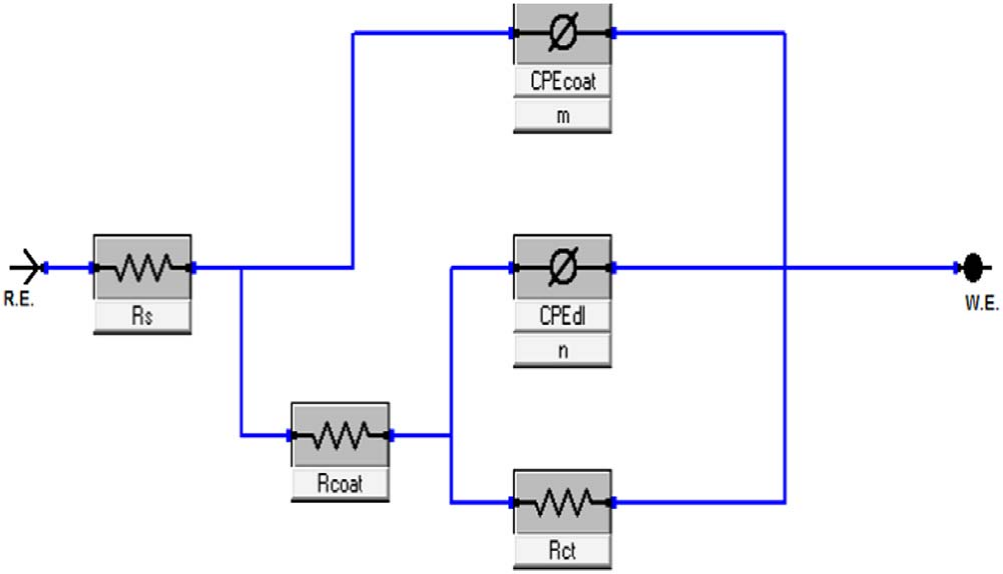
Equivalent electrical circuit to model EIS data of TEOS coated and APTES-TEOS hybrid sol–gel coated Al specimens.

The Nyquist plots of impedance data for aluminium alloy coated with single and hybrid sol–gel precursors were presented (Fig. 1). The diameter of capacitive loop represents the resistance to the corrosion [1]. The diameter of capacitive loop increased as the amount of precursors were increased. A simple model were used to fit the experimental data in the Nyquist plots for bare Al (Fig. 2) and coated Al alloy (Fig. 3). The fitting circuit was done by EChem Analyst software. Table 1 shows the list of the impedance parameters of the Nyquist plots for coated aluminium alloy of both single (TEOS) and hybrid (APTES-TEOS) sol–gel precursors. Randle׳s-CPE is used in the circuit as it fit the semicircle more accurately than Randles-Cdl circuit [2]. It was revealed from Table 1 that the value of charge transfer resistance, R_ct_ increased was higher for hybrid (APTES-TEOS) sol–gel to that of single (TEOS) precursor sol–gel. The increasing value of R_ct_ leads to an increase in percentage of inhibition efficiency as calculated from Eq. (1) [3]. Additionally, the slight changes for values of solution resistance, Rs is possibly due to the inconsistent distance between working electrode and reference electrode while conducting the experiment.

(1) $$\mathrm{IE}\;\%=\left(1-\frac{R_{\mathrm{ct}}}{R_{\mathrm{ct}}\prime }\right)\times 100\%$$

Where *R_ct_* and *R_ct_*’ are defined as the charge transfer resistance (Ω cm^2^) in absence and presence of sol–gel coatings, respectively.As a result, hybrid sol–gel coating provide a good corrosion inhibition as the percentage of inhibition efficiency (*IE* %) exceed 90%.

**Table 1:** EIS parameters for bare Al, Al coated with TEOS and Al coated with APTES-TEOS hybrid sol–gel.

Nature of the specimen	*R_s_*/Ω cm^2^	*R_coat_*/Ω cm^2^	*CPE_coat_*/µF cm^−2^	*m*	*R_ct_*/Ω cm^2^	*CPE*_*dl*_/µF cm^−2^	*n*	% *IE*
Bare Al	26.88	–	–	–	2271.67	388.9	0.757	–
TEOS sol–gel coated Al	25.77	256.98	31.58	0.832	7408.84	182.1	0.904	69.34
APTES-TEOS hybrid sol–gel coated Al	21.31	574.99	37.69	0.671	28,092.62	29.5	0.912	91.92

### 1.2 Potentiodynamic polarization

**Fig. 4:**
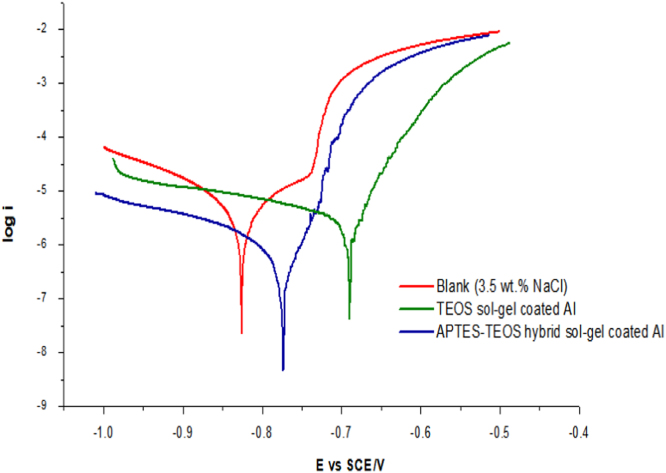
Tafel plots forbare Al, single (TEOS) and hybrid (APTES-TEOS) sol–gel coatings at 303 K.

The purpose of these investigation was to determine the influence of single or hybrid sol–gel coating precursors on the electrochemical behavior of aluminium alloy in 3.5 wt% NaCl solution based on their corrosion potential, *E_corr_*, corrosion current density, i_corr_ and the corrosion rate (Fig. 4). Table 2 shows the data of potentiodynamic polarization for aluminium alloy for coated aluminium alloy of both single (TEOS) and hybrid (APTES-TEOS) sol–gel precursors. The inhibition efficiency (*IE* %) was calculated for the aluminium alloy by using Eq. (2) [3].

(2) $$\mathrm{IE}\;\%\;=\;\frac{i_{\mathrm{corr}}{\;-\;i}_{\mathrm{corr}}\prime }{i_{\mathrm{corr}}}\times 100\%$$

Where *i_corr_* and *i_corr_*’ are the corrosion current densities (mA cm^−2^) obtained in the presence and absence of sol–gel coatings, respectively. These procedures were used for both extracts.

**Table 2:** PD parameters for bare Al, Al coated with TEOS and Al coated with APTES-TEOS hybrid sol–gel.

Nature of the specimen	*E_corr_*/mV	*i_corr_*/µA cm^−2^	*β_a_*/mV dec^−1^	*β_c_*/mV dec^−1^	*CR*/mpy	% *IE*
Bare Al	− 0.825	2.239	24	21	0.96	
TEOS sol–gel coated Al	− 0.690	0.891	39	30	0.38	60.21
APTES-TEOS hybrid sol–gel coated Al	− 0.774	0.178	53	28	0.08	92.05

The *IE*% of hybrid sol–gel coating (92.05%) was observed to be higher than single sol–gel coating (60.21%), which showed better performance in inhibit corrosion of aluminium alloy. According to literature [4], there are two types of inhibition, cathodic and anodic inhibition, which can be determined by the value of *E_corr_*, *βa* and -*βc*. From the Table 2, the value of anodic and cathodic Tafel slope constant (*βa* and − *βc*) was observed to change with the addition of the inhibitors. However, the value of *E_corr_* of both sol–gel coatings shift to positive direction indicate anodic inhibition occurs. Hence, it can be said that both sol–gel coatings showed good corrosion inhibition properties, which act as mixed type inhibitors with predominant decrease of anodic site (inhibition of metal dissolution process).

## 2 Experimental design, materials, and methods

### 2.1 Preparation of specimen

The aluminium alloy AA6061 with % weight composition of Cr 0.04, Cu 0.15, Fe 0.7, Mg 0.8, Mn 0.15 and remaining Al was cut with the dimension of 3 cm × 8 cm × 0.1 cm. Next, the plates were polished using 400, 600, 800, 1000 gritted emery papers. Next, it was degreased with methanol and washed with distilled water before and after experiment.

### 2.2 Sol gel preparation

The solution was prepared by mixing 0.1 M of tetraethyl orthosilicate (TEOS) and ethanol with stirring at room temperature for 4 h. In between, 0.1 M of hydrochloric acid (HCl) was added dropwise within the first hour. The molar ratio of TEOS: ethanol: HCl were 2:1:1. A polished aluminium plate were dipped into the mixture for 24 h [5]. For hybrid sol–gel preparation, 0.1 M TEOS, 0.1 M APTES and 0.1 M HCl were prepared with appropriate dilution using distilled water. Ethanol was used as the solvent for it form a miscible solution with the silane precursors. The 1:3 M ratio between APTES: TEOS was adopted as the suitable precursor ratio. Volumes of 15 mL of 0.1 M TEOS and 5 mL of 0.1 M APTES were mixed in a 40 mL beaker and the mixture was added with 10 mL of analytical grade ethanol. Afterwards, to drive the simultaneous hydrolysis and condensation reactions, 1 mL of 0.5 M HCl was added dropwise as the catalyst. Finally, the mixture was stirred at 30 °C for 24 h which resulted in a transparent solution [6].

### 2.3 Electrolyte

The 3.5 wt% of NaCl solution was prepared by dissolving 35 g of NaCl to 1 L distilled water and stirred until complete dissolution. The temperature of the electrolyte was maintained at room temperature (30 °C).

### 2.4 Coating procedure

Dip-coating technique, which is the most conventional coating method, was adopted as the sol–gel application process to coat the aluminium alloy substrates where they the specimens were dipped in an upright position to ensure both sides were coated. After conducting preliminary studies on dipping time, 24 h time interval, which provided the optimum and uniform coating, was selected to conduct corrosion studies. After 24 h of immersion, each aluminium alloy specimen was taken out from the sol–gel matrix and the coated samples were cured in an oven at 100 °C for 15 min to ensure the completeness of the film formation. The curing stage would result in a dense network of siloxane (–Si–O–Si–) formed on the mild steel surface. The thickness of all hybrid sol–gel coatings was typically ~ 5 μm.

### 2.5 Electrochemical measurements

Electrochemical measurements were carried out by using Gamry Instrument Reference 600 Potentiostat with EChem Analyst v5.60 software for curve fitting. For the electrochemical measurements, a 50 mL of electrodes cell assembly was used. The cell consisted of aluminium coupon with dimensions of 3 cm × 8 cm × 0.1 cm as the working electrode (WE) with exposure area 3.142 cm^2^, and the counter electrode (CE) which was platinum and saturated calomel electrode as a reference electrode (RE). Around 50 mL of electrolyte was used at room temperature for potentiodynamic polarization methods (PD) and electrochemical impedance spectroscopy (EIS), respectively. The open circuit potential (OCP) measurement was performed for 10 min to allow the stabilization for a steady state potential before PD and EIS measurements were carried out [1], [2], [3], [4].

The EIS measurement was conducted after 10 min of immersion at *E_OCP_*. The EIS were carried out with the frequency range of 0.01 Hz to 1 MHz root mean square (rms) amplitude signal of 10 mV. Potentiodynamic polarization studies were performed after 10 min immersion at open circuit potential (*E_OCP_*). The polarization was recorded by scanning the potential for range ± 250 mV with respect to open circuit potential (E_OCP_) at room temperature with the scan rate of 0.5 mV s^−1^ [1], [2], [3], [4].
